# Advances in application of swept-source optical coherence tomography angiography in diagnosis and treatment of diabetic retinopathy

**DOI:** 10.3389/fopht.2023.1116391

**Published:** 2023-02-06

**Authors:** Jinyan Zhang, Qianqian Huo, Deyu Xia, Mingfang Wang, Xiuyun Li

**Affiliations:** Affiliated Hospital of Weifang Medical University, School of Clinical Medicine, Weifang Medical University, Weifang, China

**Keywords:** swept-source optical coherence tomography angiography, diabetic retinopathy, clinical application, new developments, diagnosis, treatment and prognosis

## Abstract

Diabetic retinopathy (DR) is the most common microvascular complication of diabetes and one of the leading causes of global blinding. More attention should be paid to the diagnosis, treatment and prognosis of DR. Swept-source optical coherence tomography angiography (SS-OCTA) is a novel imaging technique presented in recent years. It can accurately present the various levels of the retina, choriocapillaris, macula, and the optic papillary microcirculation, which is new to the diagnosis and prognosis of DR. However, SS-OCTA is limited by poor fixation or severe media clouding and is susceptible to motion artefacts and segmentation errors. Future limitations need to be addressed and large prospective trials conducted to refine the relevance of SS-OCTA to DR. The present study reviews the advances in clinical application of SS-OCTA in diagnosis, treatment and prognosis of DR.

## 1 Introduction

Diabetes mellitus (DM) is a systemic disease that threatens human health. In recent years, the prevalence of DM increases annually, and the number of global patients is expected to reach 0.642 billion by 2040. Diabetic retinopathy (DR) is the major ocular manifestation of DM and one of the main causes that lead to visual impairment and blindness (1). How to assess the occurrence and development of DR and predict the prognosis with simple examination methods has long been a focus in DR research. In clinical practice, clinicians usually assess the severity of DR using multiple techniques, such as funduscopy, fundus photography, fluorescein fundus angiography (FFA), optical coherence tomography (OCT), and optical coherence tomography angiography (OCTA). FFA is an important technique used in assessment of neovascularization (NV) and non-perfusion areas (NPAs) in DR. Nevertheless, it has multiple shortcomings, such as invasiveness, time-consuming, presence of dye leakage, poor stereoscopic vision, and failure of localizing lesion depth, etc. Besides, some patients are allergic to contrast agents or not suited for examination because of renal insufficiency. All above factors limits the application of FFA in clinical practice. OCT is a non-contact, non-invasive optical diagnostic technique that can clearly depict macular morphology and retinal structure and thickness. However, it fails to show the NV lesions and NPAs. OCTA is developed based on OCT, but it provides higher resolution information of retinal and choroidal blood flow to provide more accurate microcirculation information at different levels. Nevertheless, the conventional OCTA has a limited field of view (FOV) of the retina, and the imaging quality decreases with an increase in imaging region. After many improvements over the past few years, swept-source OCTA (SS-OCTA) as the third-generation spectral domain OCT (SD-OCT) has gradually applied in diagnosis, treatment and prognosis of DR. This study provides a brief review of advances in clinical application of SS-OCTA in diagnosis and treatment of DR.

## 2 Principle and strengths of SS-OCTA

OCT was first reported by Huang et al. (2) in 1991 and gradually evolved to time-domain OCT (TD-OCT), Fourier-domain OCT (FD-OCT), and the current third-generation SS-OCTA. SS-OCTA is developed based on FD-OCT with a wavelength-swept laser source and an InGaAs-based photodetector. Multiple B-scans of the same cross-section can be acquired using SS-OCTA and a layered scanning (En-Face OCT) imaging system, and images of the erythrocytes that move in the blood flow can be captured. In this way, rapid imaging of retinal and choroidal capillary network is performed to clearly reveal the retinal and choroidal microenvironments at different levels and visualize the pathological morphology of corresponding vessels. In comparison to the first two generation OCT techniques, SS-OCTA has a faster scan speed, higher signal-to-noise (S/N) ratio and greater penetration depth. Additionally, it provides clearer and more complete images. SS-OCTA works at 100,000 A/s scan speed and 2-6 μm axial resolution, while a faster scan speed results in more intensive raster scans, which is conducive to obtaining 3D OCT data of the retina at any levels. Moreover, SS-OCTA is run with a 1,050 nm light wave, which provides the possibility of achieving accurate imaging of the microcirculation of the retina, various capillary layers of the choroid, macula and optic disc (3). It has been established that light of a long wavelength cannot be easily absorbed by fundus (3). The domain-wide widefield SS-OCTA system (TowardPi Medical Technology Ltd., Beijing, China), which is the latest product used in clinic, can obtain fundus blood flow images at a scan speed of 400,000 A/s, scan depth to the posterior segment of ≥ 6 mm and single scan range of 24 mm × 20 mm. Such images capture peripheral fundus features, which cannot be obtained by other SS-OCTA products and helps know more about the changes in the eye fundus caused by diseases.

## 3 Differences between conventional OCTA, SS-OCTA, WF-OCTA and UWF-OCTA

Conventional OCTA typically captures segments of 3 x 3 mm^2^ and 6 x 6 mm^2^ and montages these smaller scans to obtain a wider field of view to characterize NVE and other DR lesions.The SS-OCTA is available with the capability of scanning 15 x 9 mm^2^, 12 x 12 mm^2^ and even 15 x 15 mm^2^ in a single shot. These larger scan protocols reduce the scan time for montage of multiple images and provide a wider field of view, but may be more susceptible to imaging artefacts.WF-OCTA used with Extended Field Imaging (EFI), montage techniques or single shot widefield scanning can obtain a larger field of view (extending the imaging field to 60°-70°).Equipped with the ability to scan 24 x 20 mm^2^ in a single shot, the UWF SS-OCTA offers speed and efficiency benefits to assist in the evaluation of entire macular and peripapillary retinal lesions in patients with DR.

## 4 Application of SS-OCTA in diagnosis of DR

### 4.1 SS-OCTA depicts DR clinical characteristics

Recently, SS-OCTA has been gradually applied to capture clinical fundus features of DR, mainly including microaneurysms (MAs), foveal avascular zone (FAZ), vessel density (VD), retinal capillary NPAs, inter-retinal microvascular abnormalities (IRMAs) and NVs (4).

#### 4.1.1 MAs

DR patients have metabolic abnormalities due to the onset of hyperglycemia, which usually lead to apoptosis in capillary pericytes, proliferation in endothelial cells, and thickening of basement membrane. In addition, localized vasodilation concomitantly occurs in the capillaries at the zone surrounding the occluded area, which is linked with the formation of MAs. MAs are regarded as the first reliable clinical indicator for diagnosis of DR (5). MAs present as homogeneous hyperfluorescence spots on FFA (6), while with focal, dilated saccular or spindle-shaped capillaries in both the superficial and deep capillary plexuses (SCP/DCP) (7, 8),Previous research revealed that the MAs detection rate by OCTA (62%) was lower than that by FFA due to the relatively low sensitivity to the slow blood flow within some MA subtypes (7, 9, 10), although OCTA findings of MAs were similar with the histopathologic features (11).

There are many studies investigating whether SS-OCTA is superior or can be an alternative to FFA and indocyanine green angiography (ICGA). For instance, Peres et al. (12) applied En-Face SS-OCTA (segmented into SCP and DCP, sOCTA/dOCTA) and FFA to observe MAs in 19 eyes from 10 patients with diabetic macular edema (DME). They found that the average numbers of MAs on FFA, sOCTA and dOCTA were 14.5 (2-43), 9.75 (0-37.5) and 22.5 (5.5-46.5), respectively. Comparatively, the number of MAs on dOCTA was larger than that on sOCTA (P < 0.001) and FFA (P = 0.06), suggesting superior capability of dOCTA in identifying MAs in DR patients. The study of Stattin et al. (13) comparatively analyzed the capability of FFA, ICGA and SS-OCTA (sOCTA/dOCTA) in detecting MAs in 27 eyes from 17 DR patients. The results showed that the average numbers of MAs on FFA, ICGA, sOCTA and dOCTA were 33.4 (± 22), 24.9 (± 16.9), 6.5 (± 3.7), and 18.1 (± 10.5), respectively. In statistical analysis, significant difference was demonstrated in each pairwise comparison, except for the minor difference between ICGA and dOCTA (P = 0.048; 95% CI: 0.21-13.49). The authors believed that dOCTA showed a good consistency with ICGA in detecting MAs in DR patients. Thompson’s study (14) found that OCTA showed microaneurysms in 40% of patients, while Schaal’s study (15) applying SS-OCTA showed microaneurysms in 91% of cases.Zeng (16) used UWF SS-OCTA (SCP, 24 × 20 mm2) and FA for quantitative assessment of DR. The results showed that no significant differences were found between SS-OCTA and FA in assessing FAZ (area, perimeter), NPA, IRMA, NV, and only statistically significant in quantifying MA, demonstrating that FA outperformed SS-OCTA in detecting MA and denying the notion that FFA is obsolete in DR assessment. UWF SS-OCTA represents a reliable, non-invasive and quantitative imaging technique that provides both en face angiography of the entire posterior pole as in FA, and cross-sectional B-scans covering vascular flow, depicting all the information of standard structural OCT for the assessment of the microvascular system in DR, providing a potential alternative to FA in the assessment of certain aspects of DR, and as far as the detection of MA is concerned, FA has its irreplaceability. FFA has an influential role in the assessment of neovascularisation and non-perfused areas in DR, and its application is limited by the drawbacks of being invasive, time-consuming, dye leakage, poor stereoscopic vision, inability to localise the depth of the lesion, and adverse reactions to the contrast agent, UWF SS-OCTA may offer a non-invasive alternative with similar results.

Moreover, Borrelli et al. (17) applied SS-OCTA and obtained rotational 3D OCTA images to observe the morphology and related vascular conditions of 52 MAs from 20 DR patients. The major findings included: 59.6% MAs had a saccular-shape; the number of retinal layers occupied by each MA varied between 1 and 3 and the inner nuclear layer (INL) was the most frequently occupied; the number of associated vessels for each MA varied between 1-4, while 59.6% MAs were linked with 2 vessels; vessels of 38.4% and 50.0% MAs originated from the SCP and the deep vascular complex (DVC), respectively, while those of 11.6% MAs were derived from both the SCP and DVC. Considering the findings, it was believed that the structure of MAs in DR patients was complex, while 3D OCTA images could clearly show their characteristics and vascular origin.

#### 4.1.2 Macular microenvironment

The hyperglycemic state in diabetic patients predisposes to fundus hypoxia and ischemia, resulting in occlusion of retinal capillaries and subsequent formation of NPAs. NPAs tend to increase with an aggravated DR, while a broad-range retinal NPAs can lead to disorders in retinal microenvironment and impairment of visual function. Notably, DR patients will experience significant visual impairment if the macular microenvironment is involved. OCTA is capable of accurately visualizing the macular microenvironment in DR patients with manifestations mainly including interruption and deformation of the superficial and deep capillaries (even disappearance), and enlargement of FAZs, etc. (18–21).

FAZs are foveal areas free from vessels. Under normal circumstances, the fovea is free from capillaries but surrounded by a continuous capillary loop (22, 23). FAZs are important for fine vision, while the perfusion of foveal capillaries is a significant factor that influences patient vision. Research revealed that there was a large difference between the FAZs in DR patients and healthy people. The FAZs in DR patients usually increase due to the loss of vascular integrity, and the range will be enlarged with the exacerbation of DR. In clinical settings, quantitative assessment result of FAZs is likely to be a potential biomarker for macular ischemia in DR patients and can be employed to study the changes in retinal vessels (24–26).

It is well known that it is unwise to perform FFA for early screening purposes. However, UWF or WF SS-OCT has significant value in the early diagnosis of DR, and recent studies have reported the value of WF SS-OCTA in assessing the retinal microvascular system in eyes with early DR. Wang et al. (27) applied WF SS-OCTA (12 mm × 12 mm) to measure the mean perfusion area (PA) and vascular density (VD) in the superior, nasal, inferior and temporal quadrants of each circle in no-DR and mild-moderate NPDR eyes within diameter of 1 mm, 1-3 mm, 3-6 mm, 6-9 mm, and 9-12 mm. The results showed no significant difference in mean PA and VD between the groups in the central ring (1 mm) and in the wide-field scans (9 and 12 mm radius), and only in the 1-3 mm radius range, PA and VD were significantly decreased in both the upper and lower quadrants of no DR and mild-moderate NPDR. This demonstrates that WF OCTA is useful in assessing peripheral capillary perfusion in eyes with early DR. Liu et al. (28) analyzed the size, perimeter, acircularity index (AI) and VD of FAZs in 30 healthy controls (51 eyes), 42 non-proliferative diabetic retinopathy (NPDR) patients (71 eyes) and 31 proliferative diabetic retinopathy (PDR) patients (53 eyes) using SS-OCTA. In comparison to the healthy controls, significant increase was found in the size (0.38 ± 0.08 vs. 0.49 ± 0.23, P = 0.030 < 0.05), perimeter (2.58 ± 0.32 vs. 3.08 ± 0.90, P = 0.016 < 0.05) and VD (42.70 ± 5.37 vs. 34.80 ± 5.53, P = 0.000 < 0.001) of FAZs in PDR patients, while in the perimeter (2.58 ± 0.32 vs. 2.93 ± 0.58, P = 0.029 < 0.05), AI (0.71 ± 0.09 vs. 0.64 ± 0.11, P = 0.010 < 0.05) and VD (42.70 ± 5.37 vs. 37.36 ± 5.96, P = 0.001) of FAZs in NPDR patients, suggesting potential linkage between FAZs’ alterations and DR progression. Torcato Santos et al. (29) performed SS-OCTA in 105 patients with diabetes without retinopathy (DWR) or with varying degrees of retinopathy (NPDR up to ETDRS grade 53). In patients with DWR or ETDRS grades 20 - 35, retinal capillary closure was in the macular area, with predominant changes in the parafoveal retinal circulation (inner ring); while in ETDRS grades 43 - 53, retinal capillary closure was predominant in the retinal midperiphery. In addition, the combination of acquisition protocols 3 × 3 mm and 15 × 9 mm using SS-OCTA could distinguish mild NPDR (ETDRS grades 10, 20, 35) from moderate-to-severe NPDR (ETDRS grades 43-53). The findings indicated that quantitative assessment of retinal capillary closure using SS-OCTA could help identify NPDR severity in diabetes patients.

There are also some studies that assessed the accuracy and repeatability of SS-OCTA in assessment of FAZs. In the study of Mastropasqua et al. (30), two experimenters independently measured the FAZs of 64 healthy eyes using SS-OCTA. They found that most FAZs of healthy eyes were circular or nearly circular, and the size could easily be detected as (0.269 ± 0.092) mm^2^ and (0.270 ± 0.090) mm^2^, respectively. In addition, there were no significant differences between the two experimenters in terms of the coefficient of variation (CV), coefficient of repeatability (CoR), interobserver and intraobserver concordance correlation coefficient, indicating good reproducibility and repeatability of SS-OCTA in assessment of FAZs of healthy eyes. While in the study of Hirokazu Ishii et al. (22) SS-OCTA was employed to assess the FAZs of 40 healthy eyes from 22 volunteers, while Kanno Saitama macro (KSM) and Image J system were adopted to automatically measure the size of the FAZs. The results were found comparable with those measured manually, whereas automatic measurement by the software showed little dependence on the experimenter, which might be conducive to studying various retinal diseases, especially vascular retinal diseases.

#### 4.1.3 NPAs

As DR progresses, there are a series of biological processes, including apoptosis in retinal capillary pericytes, proliferation in endothelial cells, dilation in capillaries, and decrease in vascular resistance to blood flow. In this context, the dilated capillaries work as major feeders, whereas the adjacent capillaries tend to be nonperfused and forms an area known as NPA (5). Stefánsson et al. (31) explained the mechanism underlying the formation of retinal NPAs according to the principles of physics. They pointed out that the loss of pericytes led to the thinning of the retinal capillary wall and then dilation, decreasing the blood flow in the neighboring capillaries, accelerating the flow velocity and increasing the blood flow within diseased vessels, thereby reducing the blood flow in adjacent capillaries. On SS-OCTA, retinal NPAs are defined as the regions which are localized between the small terminal retinal veins and the small terminal arteries and the large proximal vessels free from capillary bed (4).

Widefield OCTA (WF-OCTA) represents the state-of-the-art examination of vascular retinal diseases including DR, and it presents huge advantages in detecting NPAs and NV lesions. Tan et al. (32) improved the performance of WF-OCTA in diagnosis of NPAs by eliminating the effect of capillaries on larger retinal vessels, and they found that OCTA performed well than FFA in analysis of NPAs due to the absence of fluorescein leakage or influence by fluorescein leakage. Sawada et al. (33) applied ultra-widefield FFA (UW-FFA, 200^°^) and WF-OCTA (acquisition protocol: 12 × 12 mm) to detect the NPAs in 58 eyes from 33 DR patients. As a result, NPAs were detected in 47 eyes by UW-FFA while in 48 eyes in WF-OCTA (sensitivity: 0.98; specificity: 0.82). WF-OCTA, therefore, was considered as applicable in clinical detection of NPAs in DR patients. In the study of Hirano et al. (34) SS-OCTA combining extended field imaging (EFI) was applied to assess the retinal vascular conditions in 37 eyes from 27 DR patients, including NPAs, NV lesion, and VD. The results revealed that the combined use of SS-OCTA and EFI obtained a larger FOV, which was 1.80 (± 0.18) fold larger than that on SS-OCTA alone. While in comparison to FFA, combination strategy showed no statistical difference in the FOV (61.2 ± 45.8 vs. 61.5 ± 55.0) with the sensitivity of 96% and the specificity of 100% in detecting NPAs. The findings indicated that the use of SS-OCTA + EFI was associated with better patient comfort than FFA.

The study of Wang et al. (35) comparatively analyzed the performance of SS-OCTA (80° × 60°) and FFA (both centered on the fovea) in quantitative assessment of NPAs in 18 diseased eyes from 11 PDR patients. SS-OCTA was segmented into SCP and DCP, and the NPAs of the full-thickness retina, SCP and DCP were detected. The median size of NPAs on FFA was 0.786 mm^2^, while that on SS-OCTA was 0.787 mm^2^ (full-thickness retina), 0.791 mm^2^ (SCP) and (0.878 ± 0.366) mm^2^ (DCP), respectively. Statistically, the median size of NPAs at the DCP level by SS-OCTA was significantly larger than that detected by FFA, demonstrating superior performance in measuring NPAs in diseased eyes of PDR patients.

There have also been some studies related to the distribution of NPAs and the significance in examination. For example, the study of Kim et al. (36) explored the relationship between microvascular parameters and DR severity using SS-OCTA in 235 eyes, including eyes from NDR, mild NPDR, moderate NPDR, severe NPDR and PDR. The microvascular parameters from the regions (3 × 3 mm^2^, 6 × 6 mm^2^ and 10 × 10 mm^2^) perpendicular to the fovea-optic disc axis were recorded. It was found that the NPAs detected in the regions with sizes of 6 × 6 mm^2^ and 10 × 10 mm^2^ were the only parameters that could be employed to distinguish between the NPDR stages. ROC curve analysis demonstrated that the NPAs from the 10 × 10 mm^2^ region performed the best in terms of classifying DR into 5 stages (NDR, mild NPDR, moderate NPDR, severe NPDR and PDR). In the meantime, cut-off values of the NPAs from the 10 × 10 mm^2^ region for grading NDR, mild from moderate NPDR, moderate from severe NPDR and severe NPDR from PDR were 3.7% (AUC: 0.91), 4.7% (AUC: 0.94), 9.3% (AUC: 0.94) and 21.4% (AUC: 0.90), respectively. It was therefore believed that the severity of retinal ischemia presented by SS-OCTA was linked with the severity of DR, while the NPAs measured from the 10 × 10 mm^2^ scan area had the highest sensitivity for determining DR severity and might bring benefits for DR staging. Wang et al. (37) obtained ultra-widefield OCTA (UWF-OCTA) images with 100° FOV in 60 eyes with DWR, NPDR and PDR and calculated the ratio of non-perfusion (RNP), the percent area of capillary nonperfusion within the FOV, in the FOV 100° image and concentric sectors encompassing 10°, 10° - 30°, 30° - 50°, and 50° - 100°. The average RNP from FOV 50° - 100° varied among three groups (DWR vs. NPDR vs. PDR: 14.6 ± 5.1% vs. 27.5 ± 7.5% vs. 41.5 ± 19.1%), while that was significantly higher than the RNP from all other sectors in each group. Additionally, the RNP from FOV 50° - 100° exhibited the highest sensitivity in differentiating DWR, NPDR, PDR and had greater diagnostic value in determining DR severity than other sectors. It is believed that the average RNP is higher in severer DR cases. In spite of the different FOV, both the above two studies proved the linkage between DR severity and the size of retinal NPAs, and the mean NPAs tend to be higher in OCTA scans with a larger scan range or in the images of the very marginal regions with more clinical significance in assessment of DR.

To observe the relationship of retinal NPAs to arteries or veins, Ishibazava et al. (38) obtained SS-OCTA (12 × 12 mm) images from 63 eyes of 44 patients with PDR or NPDR. NPAs were calculated and classified as either arterial-adjacent or venous-adjacent based on the shortest distance, while the ratio of arterial-adjacent NPAs to venous-adjacent NPAs (A/V) was computed. The results indicated that total NPAs in PDR patients were significantly greater than those in moderate NPDR patients (median: 8.93% vs. 3.49%, P < 0.01); the number of arterial-adjacent NPAs was larger than that of venous-adjacent NPAs with the A/V ratio in moderate NPDR, severe NPDR and PDR of 1.93, 1.84 and 1.78, respectively; in addition, there was a negative relationship observed between the A/V ratio and the total NPAs (r = -0.600, P < 0.0001). Collectively, these findings suggested that SS-OCTA was conducive to understanding the non-perfusion conditions in DR patients, and that NPAs were likely to be smaller when adjacent to arteries but larger when adjacent to veins.

To investigate the retinal microvasculature in DWR eyes and its association with systemic conditions of patients, Yang et al. (39) scanned 55 eyes from 30 DWR patients using UWF SS-OCTA with a range of 12 × 12 mm and recorded the NPAs, microvascular dilation and tortuosity, and the number of new vessels. 58.2% eyes presented with microvascular disorders; NPAs and microvascular dilation and tortuosity were more common in peripheral areas than central areas; and there was a significant relationship between the number of NVs and HbA1c level in the overall and peripheral areas. The findings suggested that UWF SS-OCTA was capable of detecting the microvascular changes in diseased eyes with preclinical DR, which were linked with systemic conditions of patients.

#### 4.1.4 IRMAs and NV

IRMAs and NV are proliferative changes resulting from retinal ischemia and hypoxia, and they are characteristic fundus manifestations that can distinguish between severe NPDR and PDR. In this context, it is critically important to identify IRMAs and NV lesions in clinical diagnosis.

It is reported that IRMAs are a risk factor for NV. Russell et al. (40) performed SS-OCTA and FFA in 2 PDR patients before and after receiving pan-retinal photocoagulation (PRP). After PRP, FFA demonstrated 3 NV lesions in 1 patient and 1 NV lesion in another patient; while before PRP, the 4 lesions presented as IRMAs on FFA and intraretinal tortuous vascular lesions on SS-OCTA. After 1 week, 1 month and 3 months of PRP, the lesions developed into preretinal NV with contiguous intraretinal components. Therefore, it was believed that the retinal NV in DR patients could develop from IRMAs. Additionally, early identification of IRMAs might help predict progression to PDR, and frequent monitoring of IRMAs using SS-OCTA might be conducive to early diagnosis of PDR. The study of A.Sami Memon et al. (41) observed the features of IRMAs and NV in 28 eyes (13 NPDR eyes and 15 PDR eyes) using SS-OCTA. Fifty-six aberrant vascular lesions were found, including 36 (64%) being IRMAs restrained under the internal limiting membrane (ILM) with intra retinal flow and 20 (36%) being NV lesions. Of the 20 NV lesions, 18 (90%) were in advanced stage penetrating ILM and posterior hyaloid while 2 (2%) were demonstrated only breaching ILM. Schaal’s study (15) applying SS-OCTA showed IRMA in 79% of cases and neovascularisation in 21% of cases. Khalid et al. (42) found that OCTA-B scans detected NVD in 100% with a significant flow signal of 79.5%. WF-OCTA had a detection rate of 81% for NVE.The authors held the view that SS-OCTA could be an alternative to FFA in diagnosis of DR and an important tool to predict DR progression and distinguish between IRMAs and NV lesions.

Currently, a number of studies have investigated the accuracy of SS-OCTA and other techniques in determining of IRMAs and NV. For example, Motulsky et al. (43) found that WF SS-OCTA (scan pattern: 12 mm × 12 mm) could well present the retinal NV lesions in 24 eyes of 12 PDR patients. In the study of Lu et al. (44), WF SS-OCTA (scan pattern: 12 mm × 12 mm) and SS-OCT were comparatively analyzed for their performance in detecting NV lesions in 142 eyes from 89 DR patients. The results indicated that more retinal NV lesions were detected by SS-OCTA (P < 0.05), while the combination of SS-OCT and SS-OCTA significantly increased the detection rate of NV lesions from DR eyes (P < 0.05), reducing the false positive rate and conducive to diagnosis and monitoring of PDR at follow-up. Similarly, the comparative study performed by Pichi et al. (45) analyzed the capability of WF-OCTA, UWF-FFA and ultra-wide-field color fundus photography (UWF-CP) in determining retinal NV in 82 eyes from 82 PDR patients. It was demonstrated that both the sensitivity and specificity of WF OCTA for detecting new vessels of the disc (NVDs) were 100%, whereas those of UWF FA and UWF-CP were 94.6%, 100% and 35.1%, 97.8%, respectively. It was therefore believed that WF OCTA could identify the NVDs which were not significant on UWF-CP, and it could be used as a faster and safer alternative to UWF-FA to be applied in monitoring of PDR. While in the study of Hasenin Al-khersan et al. (46) in 47 eyes from 24 PDR patients, the percentage of correct NV grading was 87.8% using SS-OCTA with B-scans while 86.2% using FFA (P = 0.92), suggesting that SS-OCTA might be a proper approach for diagnosis of NV in diabetes. Papayarnlis et al. (47) imaged 186 eyes from 93 DR patients using SS-OCTA with three segmentation protocols: (1) Vitreo-Retinal Segmentation (VRS): the lower boundary was set to posterior ILM to include the outer retinal layer (especially the SCP), and the upper boundary was set in the cortical vitreous at an appropriate to include any evident hyper-reflective structure on OCT B-Scan; (2) Outer Vitreous Segmentation (OVS): the lower boundary was set to anterior ILM and the upper boundary was set in the cortical vitreous at an appropriate depth to include any hyper-reflective structure on the OCT B-Scan; (3) Core Vitreous Segmentation (CVS): both the lower and the upper boundaries were set in the vitreous at different depth ranges to fully or partially include any hyper-reflective structure on the OCT B-Scan located within the vitreous cavity and to evaluate this structure at different vitreous depths as needed from the cortical to the core vitreous. Both the sensitivity and specificity of SS-OCTA for detecting NVDs were 100%, while those for detecting NV elsewhere (NVE) were 96.6% and 100%, respectively. It was believed that the three segmentation protocols of SS-OCTA varied but had complementary characteristics, which allowed a standardized and reproducible analysis on NVDs and NVE and were significant for research into NVDs.

### 4.2 Application of SS-OCTA in monitoring microvascular changes in choriocapillaris in DR patients

It was previously believed that DR was closely linked with impaired retinal circulation. With the result of histopathology and more examinations performed, DR patients are also found with changes in the choroid, including focal vascular dilation, filling and microaneurysms, loss and dysfunction of capillary endothelial cells (ECs), focal luminal stenosis and occlusion in capillaries. All these changes can lead to local choroidal ischemia and formation of NPAs, while the occurrence of severely impaired circulation can result in NV and fibril-membrane hyperplasia. These changes are also known as manifestations of diabetic choroidal lesions (48–53). SS-OCTA works with a light of a long wavelength, which allows a stronger penetration, a higher sensitivity and a better view of choroidal capillaries. Therefore, SS-OCTA is useful in clearly displaying the blood flow within choroidal capillaries in DR patients (54–56).

Maruko et al. (57) obtained SS-OCTA images (scan range: 12 × 12 mm, centered on the macula) of 61 eyes from 45 patients without ocular diseases (19 males and 26 females), based on which the subfoveal choroidal thickness (SCT) was measured, the ratio of the choroidal blood flow area in a slab 30 - 60 µm posterior to the retinal pigment epithelium was calculated and the corresponding choroidal blood flow pattern was analyzed. After the subtraction, SS-OCTA images of all eyes clearly revealed the middle and large choroidal blood flow; there was a significant linkage between the SCT (mean: 297 ± 61 µm) and the ratio of the choroidal blood flow area (mean: 27.3 ± 8.2%) (R = 0.738, P < 0.01). The findings suggested that the choroidal blood flow area might be an important indicator of SCT, which also meant that the changes in choroidal blood flow might lead to altered SCT thereby some functional changes. Liu et al. (28) applied SS-OCTA to measure the choroidal perfusion (CP), vascular index (CVI), vascular volume (CVV) and thickness (CT) within the radius of 3, 6, 9, and 12 mm around the fovea. In comparison to the control group, patients with NPDR and DR had higher CT levels but significantly lower CP levels. While comparing PDR with NPDR patients, the CT values in 3-6 mm (3-6 mm^2^) captures varied markedly, and the CP values within the radius of 3, 6, and 9 mm around the fovea were much lower. Notably, the degree of CP decrease was directly linked with the progression from NPDR to PDR. In addition, the mean CVI values at 9-6 mm^2^ were distinctly different between PDR and NPDR patients. However, there was only a minor difference in the CVV values at 3 mm (3 × 3 mm) captures between the control and PDR groups (0.70 ± 0.32 vs. 0.85 ± 0.41, P = 0.033). The findings suggested that the CP and CVV were significant in DR grading, while SS-OCTA could be a tool capable of providing relevant information.

Besides the choroidal flow and thickness, changes in the choriocapillary filling defects have also been investigated in diabetic patients. The study of Dai et al. (58) explored the microvascular changes in choriocapillaris (CC) by using SS-OCTA in 16 diabetic eyes from 16 DWR patients and 16 eyes from 16 non-diabetic controls. As compared to non-diabetic controls, the percentage of flow deficits (FD%) and the flow deficit (FD) sizes of CC significantly increased in the central 1.0-mm disk, central 1.5-mm rim, central 2.5-mm rim, entire 5.0-mm disk in DWR patients. This result suggested a decrease in macular choroidal perfusion in DWR patients, which might be an early indicator of otherwise clinically undetectable diabetic vasculopathy. Another study of Dai et al. (59) compared the FD% and FD size of CC within the radius of 5 mm around the fovea between DR eyes (n = 45) and normal eyes (n = 27) using SS-OCTA. The authors found that both the mean FD% (12.34 ± 4.14% vs. 8.82 ± 2.61%, P < 0.001) and mean FD sizes (2151.3 ± 650.8 μm2 vs. 1574.4 ± 255.0 μm2, P < 0.001) of diabetic eyes were 1.4 times larger than that of normal eyes, but both of them showed no significant differences between diabetic eyes from NPDR and PDR cases. It was believed that the FD% and FD size of CC could be used to quantitatively assess the choroidal perfusion in DR patients with the help of SS-OCTA system. While in the study of Gendelman et al. (54),the significance of CC FD% in DR grading was investigated in different macular regions (inner, 0.5 mm; middle, 0.5 mm - 1.5 mm; outer, 1.5 mm - 3 mm; and full-field region) of the SS-OCTA images of 160 diabetic eyes from 90 diabetic patients. The results demonstrated that the CC FD% had significant positive associations with the patient age and DR severity in each macular region, and such association was much more distinct in the two centermost regions. In addition, the CC DF% in inner, middle, outer, and full-field regions increased by 0.12 (P < 0.001), 0.09 (P < 0.001), 0.05 (P < 0.001) and 0.06 (P < 0.001), respectively, per year of age; while that increased by 0.65 (P < 0.0087), 0.56 (P < 0.0012), 0.33 (P < 0.045) and 0.36 (P < 0.018), respectively, per increase in DR severity stage. It was demonstrated that the CC flow impairment was linked with DR severity. All studied regions were significantly affected upon increasing DR severity, with the inner and middle regions being affected the most.

### 4.3 Application of SS-OCTA in DR patients after treatment

Clinical therapies for DR mainly include drug therapies, intra-vitreal injection of anti-vascular endothelial growth factor (VEGF) agents, intra-vitreal injection of dexamethasone implant (IDI), PRP, subthreshold micropulse laser (SMPL), and surgery, etc. SS-OCTA as an emerging imaging diagnostic technique has multiple strengths, such as fast scan speed, high resolution, deep probing depth, wide scan range, and function of automated quantitative analysis. It has been gradually applied in evaluation of clinical efficacy in DR eyes after treatment.

#### 4.3.1 Application of SS-OCTA in DR patients receiving anti-VEGF therapy

Couturier et al. (60) observed the changes in retinal NPAs in diabetic macular edema (DME) patients after anti-VEGF treatment by using WF SS-OCTA and UWF FA images of 10 diabetic eyes from 9 patients with NPDR or PDR, and the NPAs, microaneurysm (MA) and retinal hemorrhage at baseline and 1 month after the third anti-VEGF injection. All NPAs found on UWF FA could also be seen on WF SS-OCTA, while there were additional NPAs 29% (46/160) detected on WF SS-OCTA. One month after the third anti-VEGF injection, the mean numbers of MAs and retinal hemorrhages on UWF FA significantly decreased, and there was no reperfusion in NPAs. The findings implied that WF SS-OCTA performed better than UWF FA in detecting NPAs, and there was no reperfusion in NPAs after anti-VEGF therapy.

The study of Pongsachareonnont et al. (61) evaluated the retinal MAs and FAZs using SS-OCTA in 152 DME eyes before and after 1 month of injection of anti-VEGF agents (Aflibercept, Ranibizumab, and Bevacizumab). After 1 month of injection, the number of MAs and the areas of FAZs in SCP and DCP significantly reduced, while the changes in FAZs in SCP and DCP corresponded with changes in visual acuity. Decreased number of MAs in DME eyes might be used as a clinical indicator for short-term response to anti-VEGF therapy, and the changes in FAZs might predict visual acuity improvement after treatment. For the reduction in FAZ area following anti-VEGF treatment, Dabir (62) hypothesised that the majority of the reduction in FAZ size was due to a concomitant reduction in capillary displacement secondary to the regression of intraretinal oedema, rather than an improvement in macular perfusion. Dabir treated 24 eyes with NPDR (11 with moderate NPDR and 13 with severe NPDR) with intravitreal injections of anti-VEGF (ranibizumab) over a period of three months. Serial OCTA measurements at baseline and 1 month after three intravitreal injections of ranibizumab revealed no change in FAZ circularity either, indicating no change in the configuration of the FAZ capillary rim, and the results confirmed the mechanical displacement theory of FAZ size change rather than an ischaemic cause.A short follow-up period may not be sufficient to detect the magnitude of efficacy of different drugs by OCTA findings. Longer prospective observation periods and larger sample sizes may be needed to obtain more data to support research findings.

#### 4.3.2 Application of SS-OCTA in DR patients treated with IDI

In the study of Toto et al. (63) 28 patients with DR and DME were included and assigned to undergo SS-OCTA (a scan range of 15 × 9 mm centered on the fovea) to analyze NPAs and VD in SCP and DCP at baseline, 1, 2 and 4 months after IDI treatment. The results indicated that IDI treatment led to significant reductions in NPAs in SCP from 11.4% baseline to 6.3%, 8.1% and 10.2% at 1, 2 and 4 months after treatment, respectively. In contrast, the retinal VD in the SCP slightly increased from 35.3% baseline to 38%, 37.85% and 36.04% at 1, 2 and 4 months after treatment, respectively. No obvious changes in the NPAs and VD in the DCP were demonstrated. SS-OCTA, therefore, was considered with certain significance during follow-up in patients with IDI treatment. It was also indicated that patients undergoing IDI treatment had decreased NPAs, which might be linked with reversal of nonperfusion areas after intravitreal anti-VEGF treatment in the early period.

#### 4.3.3 Application of SS-OCTA in DR patients receiving PRP

PRP is regarded as a gold standard in treatment for severe NPDR or PDR (64). It was reported that PRP is effective in regression of neovascularization (65). In addition, PRP was also studied with effects on the thickness and flow of the retina and choroid (66, 67), whereas the conclusions are diverse.

In the study of Russell et al. (68), 20 eyes from 15 PDR patients were imaged using WF SS-OCTA (12 × 12 mm scans) and UWF FFA, and the NPAs at baseline, after 1 week, 1 month, 3 months, 6 months and 1 year of PRP were recorded. It was found that the sizes of NPAs marginally varied after 3 months and even 1 year of treatment, even in the diabetic eyes with severe ischemia at baseline. It was believed that WF SS-OCTA could be applied during follow-up of DR patients after PRP, and there was almost no change in the NPAs after treatment. Moreover, another study of Russell et al. (69) also observed the changes in NV lesions after PRP and found that the progression of regression of NV lesions after PRP could be clearly seen on both SS OCTA and UWF FFA. Additionally, the vascular changes on WF SS-OCTA were depicted in more detail, which pointed out that WF SS-OCTA might be an effective approach in diagnosis of PDR and monitoring of NV lesions after treatment. The study of Kim et al. (70) quantitatively analyzed the microvascular parameters using SS-OCTA in 27 DR patients undergoing PRP. The FAZs, macular perfusion density (PD) and vessel length density (VLD) on 3 × 3 mm en face SS-OCTA images as well as the NPAs on 12 × 12 mm en face SS-OCTA images during the follow-up period of 12 months were recorded. The PD and VLD in the SCP and DCP were decreased one month after PRP but gradually increased in the following 11 months, with the differences with baseline data statistically significant (P = 0.015 and P = 0.02, respectively). Besides, the overall NPAs exhibited a continuous downward trend after PRP treatment (P = 0.125), and the difference in PD of the SCP between baseline and 6 months post PRP was distinctly linked DR progression 12 months after PRP (OR 0.528; P = 0.025). It was inferred that there were retinal microvascular changes in DR patients undergoing PRP, and the impaired macular perfusion progressively recovered across the next 12 months. Moreover, the early treatment responses in PD may predict the long-term outcomes of PDR after PRP. There were also some studies reporting that WF OCTA might be conducive to performing targeted PRP in DR patients with comparable clinical efficacy with the conventional PRP (37, 70–73).It is generally accepted that there is little difference in FAZ evaluation between conventional OCTA and SS-OCTA, and that FAZ changes may occur in DR patients after PRP. Sabaner et al. (74) used OCTA to analyse macular microvascular changes in patients with NPDR and a large FAZ (SCP layer FAZa > 0.350 mm2) after PRP and found that baseline FAZ area was larger than 1 month and 6 months after PRP (0.416 ± 0.70, 0.399 ± 0.065 and 0.407 ± 0.066 mm2; p = 0.001 and p = 0.002), confirming that PRP affects retinal microvascular morphology in patients with NPDR and large FAZ areas. Abdelhalim’s study (75) examined 30 eyes with PDR using OCTA and equally assessed superficial and deep vessel density (VD), choroidal blood flow and FAZ area at baseline and 1 and 6 months after PRP and found a significant improvement in FAZ area after PRP (0.56 ± 0.27 vs. 0.50 ± 0.21 vs. 0.46 ± 0.2, at baseline, after 1 month and after 6 months, respectively), demonstrating that microvascular changes occur at different retinal and choroidal levels in patients with PDR significantly affected by PRP.

#### 4.3.4 SS-OCTA in the follow-up of patients with pars plana vitrectomy for DR

Russell et al. (76) imaged 31 eyes of 21 patients with diabetic tractional retinal detachments (TRDs) (including 10 eyes receiving PPV) using WF SS-OCTA. The WF SS-OCTA en face images was used to capture all areas of intrapolar TRDs and fibrovascular proliferation visualized on UWF imaging.An OCTA B scan was used to show the vasculature of preretinal membranes and to identify areas of vitreoretinal traction and posterior vitreous detachment. All clinically relevant features of diabetic TRDs were identified at baseline and assessed longitudinally after PPV using WF SS-OCTA, which showed all clinically relevant features of diabetic TRDs (vitreo-retinal traction subsidence) with no significant change in retinal perfusion status after surgery. If the media is clear and adequately fixed, WF SS-OCTA may be the only imaging modality required for the diagnosis and longitudinal assessment of diabetic TRDs.

## 5 Causes and solutions for artifacts in SS-OCTA

The most common causes of artifacts are projection and segmentation errors, in addition to optical aberrations, media opacities, movement or blinking, thresholds or other limiting factors. Borrelli (77) first described the presence of eyelashes artifacts in UWF SS-OCTA images and areas of false positive hypoperfusion secondary to eyelashes artifacts. Podkowinski et al. (78) showed that SS-OCTA images revealed artifacts caused by diabetic macular oedema in approximately 25% of the eyes analysed. In addition, the number of artifacts detected varied between devices, with SSPiM detected more frequently with the Plex Elite 9000 than with the Optovue OCT-A. Li (79) quantitatively compared the clinical performance of five OCTAs, AngioVue™, AngioPlex™, Spectralis OCTA, AngioScan and SS-OCTA Angio™, with AngioVue providing images with the highest effective vascular visibility and the least motion artifacts.

As OCTA and a wider range of scanning modalities become more common in DR clinical trials, it has been found that PDR eyes using WF OCTA often show motion artefacts and segmentation errors, particularly in DR patients with DME, NV, pigment epithelial detachment (PED) or anterior retinal membrane (ERM). correction of segmentation is essential to differentiate IRMA from NVE, which is of early diagnosis of PDR, and segmentation errors should be noted and manually corrected accordingly to avoid misinterpretation of the images. Cui (80) assessed WF SS-OCTA image quality, artifacts and segmentation errors in DR and investigated the main factors affecting image quality, concluding that higher motion artifact score (MAS) would reflect more artifacts and lower image quality, and that DR and The severity of dry eye was the main factor influencing MAS (p<0.05). OCTA images with higher MAS scores limited the capability to visualise fine capillary and DR features.

How to reduce the modifiable artifacts of the WF SS-OCTA system: (1) Constantly adjust the focus so that the retinal vessels are clearly visible during prolonged scanning. manual adjustments is also a method if the patient is poorly positioned,with hyperopia or high myopia (2) keep the head position stable to avoid motion artifacts; (3) the patient can be asked to rotate the eyes or subtly adjust the eye alignment by moving the mandibular rest setting to reduce the effect of small vitreous clouding on image quality; (4) dilation, use of a gaze lamp, rest between scans, during scanning blinking and the application of artificial tears also Contribute to obtain better OCTA images.

## 6 Summary

SS-OCTA provides a technique with multiple strengths (e.g., non-invasiveness, high resolution, fast scan speed and wide-range FOV) and good performance to quantitatively analyze the retinal and choroidal vasculature and thickness. It is expected to be used in clinical monitoring of progression, treatment response and prognosis of DR as an objective detection tool. The present SS-OCTA equipment is limited by poor fixation or severe media clouding, and in addition WF OCTA often suffers from motion artifacts and segmentation errors in imaging PDR eyes, which will need to be continuously eliminated in the future to obtain better quality OCTA images. Intraoperative WF SS-OCTA imaging is not yet available to determine the appropriate surgical flat between the vitreous cortex and retina for PPV surgery, and whether SS-OCTA will reduce operative time and intraoperative complications remains unanswered. There is a relative lack of follow-up studies after DR treatment and more large prospective trials using a standardised WF SS-OCTA imaging protocol are needed to determine whether treatment affects retinal and choroidal perfusion in patients with DR and to define its clinical application value.

## Author contributions

JYZ, QQH, and XYL contributed to conception and design of the study. JYZ organized the database. QQH performed the statistical analysis. JYZ wrote the first draft of the manuscript. DYX, MFW, QQH, and XYL wrote sections of the manuscript. All authors contributed to manuscript revision, read, and approved the submitted version.
