# Supplementary material for: Advances in application of swept-source optical coherence tomography angiography in diagnosis and treatment of diabetic retinopathy
Source: Front Ophthalmol (Lausanne). 2023 Feb 6;3:1116391. doi: 10.3389/fopht.2023.1116391 (PMC11182126; doi:10.3389/fopht.2023.1116391)
Supplement: Supplementary file 1 [file Table_1.docx]

| Order of questions | Content of the question | Our response | Location of modifications |
| --- | --- | --- | --- |
| 1 | include SS-OCTA and conventional OCTA images as one or more Figures for comparison especially if there are diabetic retinopathy samples | Thompson's study ^[[14](#_ENREF_14)]^ found that OCTA showed microaneurysms in 40% of patients, while Schaal's study ^[[15](#_ENREF_15)]^ applying SS-OCTA showed microaneurysms in 91% of cases.  Schaal's study ^[[15](#_ENREF_15)]^ applying SS-OCTA showed IRMA in 79% of cases and neovascularisation in 21% of cases. Khalid et al. ^[[42](#_ENREF_42)]^found that OCTA-B scans detected NVD in 100% with a significant flow signal of 79.5%. WF-OCTA had a detection rate of 81% for NVE.  there are not many relevant articles on each indicator in the article, it is not yet possible to conduct a valid data analysis, and we will increase the literature reading for further revision at a later stage. | Page 3、Page 7（lines 105-107,283-286） |
| 2 | After SS-OCTA (especially WF and UWF) using in DRP assessment, is the FFA out of date? | Zeng[16]used UWF SS-OCTA (SCP, 24 × 20 mm2) and FA for quantitative assessment of DR. The results showed that no significant differences were found between SS-OCTA and FA in assessing FAZ (area, perimeter), NPA, IRMA, NV, and only statistically significant in quantifying MA, demonstrating that FA outperformed SS-OCTA in detecting MA and denying the notion that FFA is obsolete in DR assessment. | Page 3（lines 106-119） |
|  | In the general evaluation of DRP, can you indicate the advantages and disadvantages of FFA and SS-OCTA, if any? | UWF SS-OCTA represents a reliable, non-invasive and quantitative imaging technique that provides both en face angiography of the entire posterior pole as in FA, and cross-sectional B-scans covering vascular flow, depicting all the information of standard structural OCT for the assessment of the microvascular system in DR, providing a potential alternative to FA in the assessment of certain aspects of DR, and as far as the detection of MA is concerned, FA has its irreplaceability. FFA has an influential role in the assessment of neovascularisation and non-perfused areas in DR, and its application is limited by the drawbacks of being invasive, time-consuming, dye leakage, poor stereoscopic vision, inability to localise the depth of the lesion, and adverse reactions to the contrast agent, UWF SS-OCTA may offer a non-invasive alternative with similar results. |  |
| 3 | Early diagnosis is really important. Everyone knows that it is not wise to perform FFA for early screening purposes. However, UWF or WF SS-OCT may be valuable in the early period. | It is well known that it is unwise to perform FFA for early screening purposes. However, UWF or WF SS-OCT has significant value in the early diagnosis of DR, and recent studies have reported the value of WF SS-OCTA in assessing the retinal microvascular system in eyes with early DR. Wang et al ^[[27](#_ENREF_27)]^ applied WF SS-OCTA (12 mm × 12 mm) to measure the mean perfusion area (PA) and vascular density (VD) in the superior, nasal, inferior and temporal quadrants of each circle in no-DR and mild-moderate NPDR eyes within diameter of 1 mm, 1-3 mm, 3-6 mm, 6-9 mm, and 9-12 mm. The results showed no significant difference in mean PA and VD between the groups in the central ring (1 mm) and in the wide-field scans (9 and 12 mm radius), and only in the 1-3 mm radius range, PA and VD were significantly decreased in both the upper and lower quadrants of no DR and mild-moderate NPDR. This demonstrates that WF OCTA is useful in assessing peripheral capillary perfusion in eyes with early DR | Page 4（lines 146-156） |
| 4 | Can you specify exactly the conventional OCTA, SS-OCTA, WF-OCTA and UWF-OCTA scanning field sizes, and differences? | Conventional OCTA typically captures segments of 3 x 3 mm^2^ and 6 x 6 mm^2^ and montages these smaller scans to obtain a wider field of view to characterize NVE and other DR lesions.The SS-OCTA is available with the capability of scanning 15 x 9 mm^2^, 12 x 12 mm^2^ and even 15 x 15 mm^2^ in a single shot. These larger scan protocols reduce the scan time for montage of multiple images and provide a wider field of view, but may be more susceptible to imaging artefacts.WF-OCTA used with Extended Field Imaging (EFI), montage techniques or single shot widefield scanning can obtain a larger field of view (extending the imaging field to 60°-70°).Equipped with the ability to scan 24 x 20 mm^2^ in a single shot, the UWF SS-OCTA offers speed and efficiency benefits to assist in the evaluation of entire macular and peripapillary retinal lesions in patients with DR. | Page 2（lines 66-75） |
| 5 | There is not much difference between conventional OCTA and SS-OCTA in terms of FAZ evaluation. Add that FAZ change may occur in some patient groups after PRP | It is generally accepted that there is little difference in FAZ evaluation between conventional OCTA and SS-OCTA, and that FAZ changes may occur in DR patients after PRP. Sabaner et al ^[[75](#_ENREF_75)]^ used OCTA to analyse macular microvascular changes in patients with NPDR and a large FAZ (SCP layer FAZa > 0.350 mm2) after PRP and found that baseline FAZ area was larger than 1 month and 6 months after PRP (0.416 ± 0.70, 0.399 ± 0.065 and 0.407 ± 0.066 mm2; p = 0.001 and p = 0.002), confirming that PRP affects retinal microvascular morphology in patients with NPDR and large FAZ areas. Abdelhalim's study ^[[76](#_ENREF_76)]^ examined 30 eyes with PDR using OCTA and equally assessed superficial and deep vessel density (VD), choroidal blood flow and FAZ area at baseline and 1 and 6 months after PRP and found a significant improvement in FAZ area after PRP (0.56 ± 0.27 vs. 0.50 ± 0.21 vs. 0.46 ± 0.2 , at baseline, after 1 month and after 6 months, respectively), demonstrating that microvascular changes occur at different retinal and choroidal levels in patients with PDR significantly affected by PRP. | Page 11（lines 451-462） |
| 6 | Add "Key Summary Points" with editorial permission | We have added to the“Abstract”section, which can be found on page 3 | Page 3（lines 9-18） |
| 7 | Add future expectation or direction of SS-OCTA to the Summary | We have added to the “summary”section, which can be found on page 12 | Page 12（lines 504-516） |
| 8 | " PMID: 33235428 DOI: 10.2147/OPTH.S270410 " in the study you mentioned, it seems that only one dose (!) of post-injection evaluation was performed. | A short follow-up period may not be sufficient to detect the magnitude of efficacy of different drugs by OCTA findings. Longer prospective observation periods and larger sample sizes may be needed to obtain more data to support research findings. | Page 10（lines 410-412） |
|  | Here, there is a serious study showing that OCTA can be compared with functional vision (analysis of microperimetry) after 3 loading doses! " PMID: 35322868 DOI: 10.1007/s11845-022-02979-y " I want to ask using with this study: OCTA also serves as correlation with microperimetry analysis in terms of functional vision evaluation in DRP patients. Could it be true? | I apologise for not being able to give more professional answers to the experts' questions |  |
| 9 | create an "artifact problems" sub-title. | We have created the title "4. Causes and solutions for artifacts in SS-OCTA" and have answered the questions posed by the experts | Page 11-12（lines 474-502） |
|  | Talk about SS-OCTA artifacts and solutions that may occur in DRP patients |  |  |
|  | whether the artifact problems increase in parallel as the scanning area increases. |  |  |
