# Supplementary material for: Advances in application of swept-source optical coherence tomography angiography in diagnosis and treatment of diabetic retinopathy
Source: Front Ophthalmol (Lausanne). 2023 Feb 6;3:1116391. doi: 10.3389/fopht.2023.1116391 (PMC11182126; doi:10.3389/fopht.2023.1116391)
Supplement: Supplementary file 2 [file Table_2.docx]

| Order of questions | Content of the question | Our response | Location of modifications |
| --- | --- | --- | --- |
| 1 | why FAZ area would be reduced after anti-VEGF therapy? | For the reduction in FAZ area following anti-VEGF treatment, Dabir ^[[63](#_ENREF_63)]^ hypothesised that the majority of the reduction in FAZ size was due to a concomitant reduction in capillary displacement secondary to the regression of intraretinal oedema, rather than an improvement in macular perfusion. Dabir treated 24 eyes with NPDR (11 with moderate NPDR and 13 with severe NPDR) with intravitreal injections of anti-VEGF (ranibizumab) over a period of three months. Serial OCTA measurements at baseline and 1 month after three intravitreal injections of ranibizumab revealed no change in FAZ circularity either, indicating no change in the configuration of the FAZ capillary rim, and the results confirmed the mechanical displacement theory of FAZ size change rather than an ischaemic cause. | Page 10（lines 402-410） |
| 2 | please change “agents” to a specific word | Modified, see page 10 | Page 10（lines 422-423 ） |
| 3 | add some info about SS-OCTA after diabetic vitrectomy. | We have added the title "3.3.4 SS-OCTA in the follow-up of patients with pars plana vitrectomy (PPV) for DR" on page 11 | Page 11（lines 463-473） |
| 4 | add the limitations of current technology and future directions for its improvements. | We have added the title "5.summary”on page 12 | Page 12（lines 504-516） |
